# Circadian regulation of the PERIOD 2::LUCIFERASE bioluminescence rhythm in the mouse retinal pigment epithelium-choroid

**Published:** 2010-12-07

**Authors:** Kenkichi Baba, Anamika Sengupta, Manfredi Tosini, Susana Contreras-Alcantara, Gianluca Tosini

**Affiliations:** Neuroscience Institute, Morehouse School of Medicine, Atlanta, GA

## Abstract

**Purpose:**

The retinal pigment epithelium (RPE) plays an important role in the maintenance of the health and function of photoreceptors. Previous studies have shown that the RPE is also involved in the regulation of disc shedding, a process that is vital for photoreceptor health. This process has been shown to be under circadian control, although the mechanisms that control it are poorly understood. The aim of the present study was to investigate Period 2 (*Per2*) mRNA levels in the mouse RPE in vivo, and to determine whether the cultured RPE-choroid from PERIOD 2::LUCIFERASE (PER2::LUC) knockin mice expresses a circadian rhythm in bioluminescence.

**Methods:**

*Per2* mRNA levels were measured using real-time quantitative RT–PCR, and bioluminescence was measured in PER2::LUC knockin mice using a Lumicycle®.

**Results:**

*Per2* mRNA levels in the RPE-choroid show a clear circadian rhythm in vivo. A circadian rhythm in PER2::LUC bioluminescence was recorded from cultured RPE-choroid explants. Light exposure during the subjective night did not cause a circadian rhythm phase-shift of PER2::LUC bioluminescence. Finally, removal of the suprachiasmatic nuclei of the hypothalamus did not affect the bioluminescence circadian rhythm in the RPE-choroid.

**Conclusions:**

Our results demonstrate that the RPE-choroid contains a circadian clock, and the regulation of this circadian rhythm resides within the eye. These new data indicate that it may be useful to design studies with the aim of elucidating the molecular mechanisms responsible for the regulation of the rhythmic event in the RPE.

## Introduction

The mammalian retina contains an independent circadian clock system that controls many retinal functions. Emerging experimental data also indicate that the circadian organization of the mammalian retina is comparatively complex [1]. Earlier studies have suggested that the circadian clock controlling retinal melatonin synthesis in mammals is likely to be located in the photoreceptors [2-4], as has been reported for non-mammalian vertebrates [5,6]. However, a recent study using the PERIOD 2::LUCIFERASE (PER2::LUC) fusion protein in mice [7] has reported the presence of a robust and long-term PER2::LUC circadian rhythm in cultured retina that lack photoreceptors, thereby demonstrating the presence of a circadian clock in the inner retina [8]. Additional studies with PER2::LUC mice have also shown that in the intact retina, the circadian rhythm in PER2::LUC bioluminescence can be phase-shifted by light, light exposure-induced phase delay in the early subjective night, and phase advance in the mid-subjective night [9]. Blockage of the dopamine receptor 1 (DR1) receptor prevented, albeit not fully, the light-induced phase-shift, suggesting that this phenomenon is mediated through the DR1 [9]. A key role for dopamine in the entrainment of the circadian rhythms of clock genes in the mouse retina was also suggested in a previous study [10].

The retinal pigmented epithelium (RPE) plays a key role in the regulation of disc shedding in photoreceptors, which is a rhythmic phenomenon controlled by the circadian clock [11-14]. The circadian rhythm in disc shedding persists in rats after the suprachiasmatic nuclei (SCN) of the hypothalamus—the master circadian clock in mammals—has been destroyed [15], indicating that the control of this rhythm resides within the eye. However, a previous study also showed that disc shedding in rats cannot be re-entrained (synchronized) to new light-dark cycles once the optic nerve has been severed, suggesting that a central component may be involved in its regulation [13]. Finally, it has been reported that cultured human RPE cells can express a circadian rhythm in adenylyl cyclase activity [16] and in Bmal1-luciferase [17].

The aim of the present study was to investigate Period 2 (*Per2*) mRNA levels in the mouse RPE in vivo, and to determine whether the cultured RPE-choroid obtained from PER2::LUC knockin mice expresses a circadian rhythm in bioluminescence.

## Methods

### Animals

PER2::LUC knockin mice (n=120) were used in this study. All procedures were approved by the Institutional Animal Care and Use Committee (IACUC) at the Morehouse School of Medicine. Mice were raised at the Morehouse School of Medicine in a 12 h:12 h light-dark (LD) cycle with lights on from 06:00 to 18:00 h EST. The light was supplied with fluorescent tubes (F34CW-RS-WM-ECO: General Electric, Fairfield, CT), and its intensity ranged from 100 to 150 µW/cm^2^ at cage level.

### Tissue culture preparation

Mice were first anesthetized by using CO_2_ and then sacrificed by cervical dislocation. The eyes were removed, placed in iced Hank’s salt solution, and then dissected. After the vitreous humor was removed, the retina and RPE-choroid cup were separated. The eye cup containing the RPE-choroid was flattened using four small corner cuts, and then placed on the culture membrane (Millicell-CM, PICM030–50; Millipore, Billerica, MA) in a 35 mm Petri dish with 1.2 ml of 199 medium (02732; Cambrex, Walkersville, MD) containing 10 mm HEPES (pH 7.2), 0.1 mM D-Luciferin K salt (Molecular Imaging Products, Bend, OR), penicillin/streptomycin cocktail (2.5 ml ⁄ l,15140–122; Gibco, Grand island, NY), and B27 (2%;17504–044; Gibco). RPE-choroid explants were placed—RPE cell layer up—on the culture membrane. Dishes were well sealed and kept at a temperature of 37 °C. A similar procedure was used for the retina. The tissue cultures were prepared under the fluorescent tubes at zeitgeber time (ZT) 3 to ZT10. It is conventional to divide the 24 h LD cycle into 24 h ZT units and indicate the lights-on time as ZT0 and the lights-off time as ZT12.

### Measurement and analysis of bioluminescence

The bioluminescence emitted by the retina and RPE-choroid or the choroid was measured for 1 min at 10-min intervals with a microplate luminometer equipped with photomultiplier tubes (Lumicycle®; Actimetrics, Wilmette, IL). Bioluminescence recordings were detrended using a 24 h moving average-subtraction method and smoothed by a 2 h moving average. Daily peaks were identified by Origin® software (Origin Lab, Northampton, MA) and the first peak in the smoothed data after 24 h in vitro was used as a phase marker [6,18]. The circadian period was determined from the slope of a linear regression line fitted to three–four consecutive circadian peaks before any pulses. The amplitude of the circadian rhythm was defined as the difference between the peak and trough values in the detrended time series data, and the relative amplitude was obtained as a percentage of the amplitude between the peaks before and after the pulse with respect to the time stimulation given.

### Light pulse

Dishes containing the RPE-choroid were gently removed from the Lumicycle®, placed in an identical incubator at the same temperature (37 °C), and exposed to fluorescent light (800 µW/cm^2^ for 10 min, General Electric 1010; General Electric). Then, they were returned to the Lumicycle® and placed in the same positions that they occupied before the pulse. Control samples were treated the same as the experimental samples except they were not exposed to fluorescent light. The entity phase-shift (in hour) was calculated by comparing the regression lines fitted to the circadian peaks before and after the pulse [18]. For further statistical analysis, the phase-shifts in individual culture dishes were averaged in 6 h bins with intervals of CT0–6, CT6–12, CT12–18, and CT18−24.

### RNA isolation and real-time quantitative Reverse Transcription Polymerase Chain Reaction (RT–PCR)

The retina and RPE were gently peeled from the posterior part of the eye cup, and placed in a tube containing TRIzol (Invitrogen, Carlsbad, CA). The total RNA was reverse-transcribed into cDNA at 48 °C for 37 min using random hexamers and murine leukemia virus reverse transcriptase enzyme (Reverse Transcription Reagent Kit; Applied Biosystems, Foster City, CA). One μg of RPE and retina cDNA was amplified by real-time quantitative RT–PCR using iQ iCycler (BioRad Laboratories, Hercules, CA), quantiTech SYBER green PCR master mix (Qiagen, Valencia, CA), and specific primers. Amplification efficiencies were indicated by the linearity of the logarithmic algorithm and determined using Pearson’s correlation coefficient and the linearity of the curve. The expression level of *18S* rRNA was used to normalize the expression of *Per2* mRNA in these tissues. Finally, the difference in the expression of *Per2* at different time points (C_T_) relative to C_T_4 were determined as 2^–(∆CT sample - ∆CT calibrator)^ [19]. The primers used for the experiment were *Per2* forward: 5′-TAG AAT CCC TCC TGA GAA GA-3′; reverse: 5′-GAG AAT AAT CGA AAG GCT GT-3′ (218 bp); 18S forward: 5′-AAT TCC GAT AAC GAA CGA GA-3′; reverse: 5′-ATC TAA GGG CAT CAC AGA CC-3′ (141 bp).

### SCN lesion and behavioral analysis

Mice were anesthetized with ketamine (80 mg/kg) and xylazine (16 mg/kg) and placed in a stereotaxic apparatus (David Kopf Instruments, Tujunga, CA). A small opening at 0.2 mm anterior to the bregma was made in the mouse skull with a 0.5 mm diameter drill after removing the hair and skin at the site. Lesions were made through the epoxylite-coated stainless electrode with 0.1 mm exposure (Rhodes Medical Instruments Inc., Summerland, CA) placed at 0.2 mm anterior, 0 mm medio-lateral, and 5.8 mm ventral relative to the bregma. Each mouse received a direct current of 1.5 mA for 15 s, using a specific lesion maker (Ugo Basile, Comerio, Italy). In sham-operated mice, an electrode was also inserted at the SCN site for 15 s without giving electrical current. After making the lesion, we closed the small opening in the skull with bone wax (World Precision Instruments, Sarasota, FL) and closed the surgical site with wound clips (Becton Dickinson, Baltimore, MD) and 16 mm needle silk sutures (CP Medical, Portland, OR). During the 48 h following the SCN lesion, we administered analgesics (0.1 mg/kg buprenorphine at 12 h intervals). The mice were placed on an electrical heater (37 °C) until they recovered from anesthesia. Then, the mice were placed in a 12 h:12 h light-dark cycle, and observed for 3–4 weeks to ensure full recovery. Finally, the mice were placed in constant darkness and the locomotor activity rhythm was recorded using a computerized system (Clock Lab; Actimetrics, Wilmette, IL). To confirm arrhythmicity in the locomotor activity, rhythm locomotor activity records were analyzed with the χ^2^ periodogram. Mice that showed an arrthymic behavior (i.e., mice in which the SCN was successfully lesioned) were used for the experiment. After screening behavioral rhythmicity for 3 weeks, all mice were placed in a 12 h:12 h light-dark cycle for 2 weeks until culture preparation. To confirm the anatomic location of the lesion, the brains (after eyes were sampled) were removed, frozen, stored at −80 °C, and then sectioned at the level of the SCN using a cryostat (Microm, Walldorf, Germany). The sections (20 μm thick) were then fixed with 4% paraformaldehyde and stained with cresyl violet, and the lesion site was confirmed using a light microscope.

## Results

### In vivo *Per2* mRNA expression in the RPE and retina

**Figure 1:**
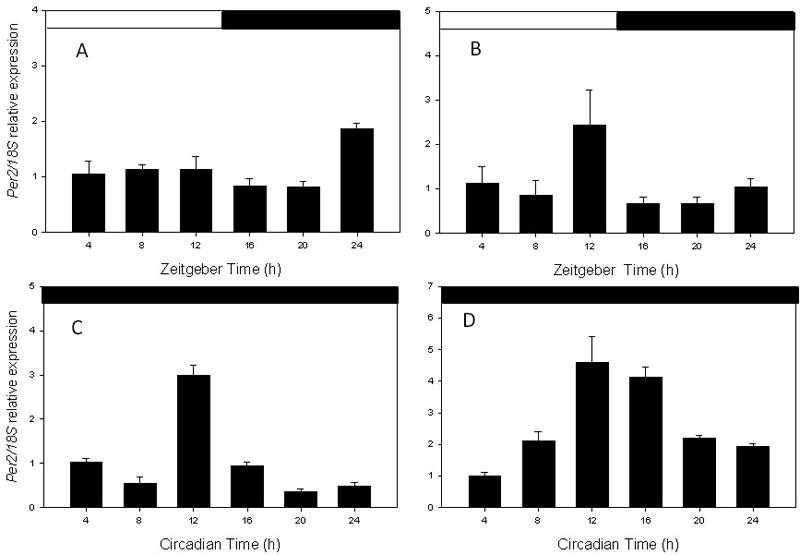
*Per2* circadian expression in retina and retinal pigment epithelium (RPE). **A**: RPE in Light/Dark cycle. **B**: Retina in Light/Dark cycle. **C**: RPE in constant darkness. **D**: Retina in constant darkness. In both tissues, the *Per2* mRNA levels showed a significant circadian rhythm (one-way ANOVA, p<0.05 in all cases). Each point represents the mean±SEM (n=3–7).

To determine the expression pattern of *Per2* mRNA in the RPE and retina, we collected RPE and retina samples at four-hour intervals under LD conditions and on the third day in constant darkness (DD) starting at CT 0. *Per2* mRNA levels were then measured using real-time quantitative RT–PCR. As shown in Figure 1, *Per2* mRNA levels in both tissues showed a clear circadian rhythm (one-way ANOVA, p<0.05 in all cases) in LD and DD (Figure 1A-D). Interestingly, *Per2* mRNA in the RPE peaked at ZT 24–0 (i.e., at the transition between dark and light) and at ZT12 in DD (i.e., at the transition between light and dark and light) in LD.

### Circadian rhythms in Per2::LUC bioluminescence in cultured RPE and retina

**Figure 2:**
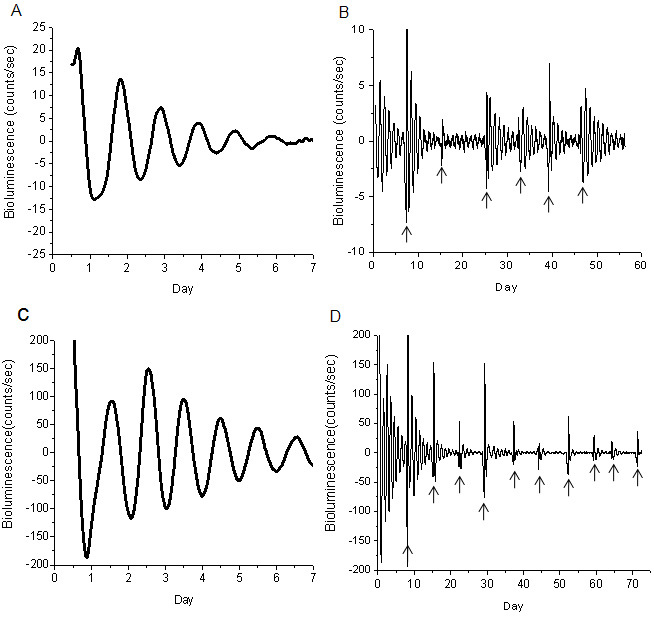
PER 2::LUC bioluminescence rhythms in RPE-choroid and retina. **A**: Representative example of PER2::LUC rhythms in RPE-choroid. **B**: The circadian rhythms of RPE-choroid persisted more than 50 days. **C**: Representative example of PER2::LUC rhythm in retina. **D**: The circadian rhythms of retina persisted more than 70 days. Although the circadian rhythm in the bioluminescence damped out after 6–7 days, a medium exchange could reinitiate the circadian rhythm in these cultures. The arrows indicate the days on which the medium in the culture was replaced.

Figure 2 shows representative results obtained by culturing the RPE-choroid and retina of PER2::LUC mice. Our data showed that the PER2::LUC bioluminescence rhythm in isolated RPE-choroid and retina explants had a robust circadian rhythm (Figure 2A,C) that persisted, in some cases, for more than fifty days (Figure 2B,D). The average circadian peak phase of PER2::LUC bioluminescence in the retina was ZT 12.40±0.32, and in the RPE, it was ZT 16.54±0.20. The average circadian period was 24.32±0.15 h (mean± Standard Error of the Mean [SEM], n=53) and 23.88±0.12 h (mean±SEM, n=81), respectively, for the retina and the RPE.

### Effect of light pulse on the PER2::LUC bioluminescence rhythm

**Figure 3:**
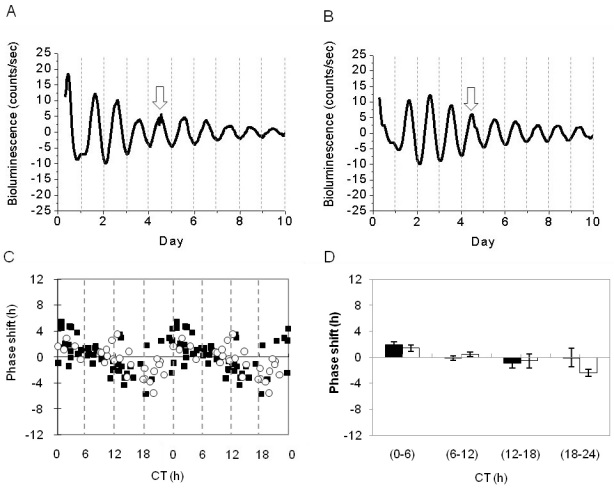
Light responsiveness of PER2::LUC bioluminescence rhythm in retinal pigment epithelium (RPE)-choroid. **A**: The light pulse was given at Day 4 of PER2::LUC rhythms. **B**: The control pulse was given at Day 4 of PER2::LUC rhythms. Arrows indicate the times of the treatment. **C**: The double-plotted phase response curve to light stimuli in the PER2::LUC bioluminescence rhythm in the RPE-choroid. Black squares indicate control and white circles indicate the samples that received the light pulse. **D**: Data were divided into 4 bins in 6 h intervals for statistical analysis. No significant differences were observed between the control (black bars) and the experimental (white bars) groups (two-way ANOVA, p>0.1).

RPE-choroids were exposed on the third day of culture to 10 min of fluorescent light (intensity, 800 µW/cm^2^) at several different phases of the circadian cycle to obtain a phase-response curve to the light stimuli; then, the bioluminescence was continuously measured for an additional five days (Figure 3A,B). Light did not induce a significant phase-shift in the PER2::LUC circadian rhythm at any of the times investigated (n=28, experimental; n=53, control; two-way ANOVA, p>0.1; Figure 3C,D). No change in the amplitude or period of the circadian rhythm was observed after the light pulse (amplitude, 64.1±2.63 versus 70.6 ± 5.02; period, 23.7±0.15 versus 23.3±0.12; *t*-tests, p>0.1 in all cases).

### Effect of the SCN lesion on the PER2::LUC bioluminescence rhythm

**Figure 4:**
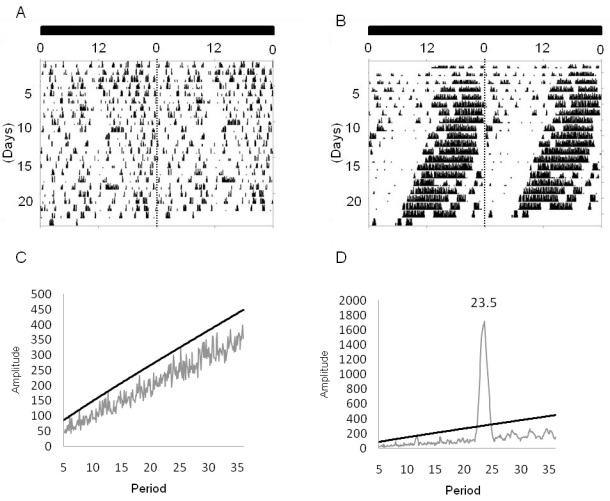
Circadian rhythm of locomotor activity in SCN lesioned mice. **A**: Representative actogram of a mouse that received SCN lesion. **B**: A mouse that received a sham lesion. **C**: The periodogram analysis (χ2) indicated that the locomotor activity is no longer rhythmic after the SCN has been lesioned. **D**: It is still rhythmic in the sham-lesioned mice.

**Figure 5:**
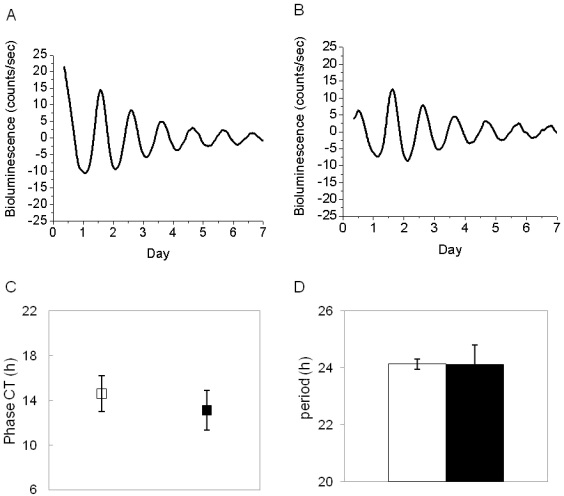
Effect of SCN lesion on the RPE-choroid circadian rhythm. **A**: Representative example of the RPE-choroid PER2::LUC bioluminescence rhythm in a sham-lesioned mouse **B**: In a SCN-lesioned mouse. **C**: Removal of the SCN did not affect the phase of the PER2::LUC RPE-choroid circadian rhythm (white square represents sham-lesioned mice; black square represents SCN-lesioned mice) **D**: Period of the circadian expression (white bars represents sham operated mice; black bars represents SCN-lesioned mice; *t*-tests, p>0.1 in both cases).

The SCN lesion was induced in PER2::LUC mice (n=6), after which the locomotor activity was monitored for three weeks in constant darkness to verify arrhythmicity in their activity (Figure 4). Once this arrhythmicity was verified, the mice were returned to a 12 h:12 h light-dark cycle for ten days and then sacrificed in the middle of the light phase (ZT 4–6). The eyes were removed and the RPE-choroid explants were cultured as previously described in the method section. The brains were also removed to verify the absence of SCN. Control animals (n=12) received a sham lesion and were treated in the same manner as the mice that received the SCN lesion. RPE-choroid explants from SCN-lesioned mice showed a clear circadian rhythm in PER2::LUC bioluminescence with a phase, amplitude, and free-running period the same as those of sham-operated mice (*t*-tests, p>0.1, in all cases, Figure 5)

## Discussion

In this study, we report that *Per2* mRNA levels show a circadian rhythm in the mouse RPE in vivo and demonstrate that the RPE-choroid of PER2::LUC knockin mice expresses a robust circadian rhythm in bioluminescence. The circadian rhythm in PER2::LUC bioluminescence cannot be phase-shifted by a light pulse, suggesting that the RPE-choroid is not directly photosensitive. Finally, our investigation suggests that lesions of the SCN do not affect the circadian rhythm in the RPE-choroid or the phase of the rhythm, so the control of this circadian rhythm resides within the eye.

Previous studies have reported that *Per2* mRNA levels in the mouse retina peak around ZT10 in LD and DD (8) and our data are consistent with this finding (Figure 1B,D). The change in the peak time for the *Per2* mRNA levels between LD and DD observed in the RPE (Figure 1A,C) is difficult to explain. Previous studies have established that dopamine may play a key role in the entrainment of the retinal circadian clock system [7,9,10,20], and it is well known that dopamine levels are no longer rhythmic in C57 mice (the same strain of mice used in this study) after two days in DD [20-22]. Therefore, we hypothesize that the dopamine released from dopaminergic neurons in the inner retina is the signal used by the RPE to synchronize its circadian clock with the environmental light cycles, and that once that this signal is lost, the circadian system in the RPE will reset to a different phase. Further studies using C3H mice (a mouse strain in which the dopamine rhythm is preserved in DD) are required to investigate this hypothesis.

Previous studies have shown that many ocular structures express a circadian rhythm in PER2::LUC bioluminescence. For example, Yoo et al. [7] reported that PER2::LUC bioluminescence is rhythmic in the cornea and Ruan et al. [8,9] demonstrated that the retina also expresses a clear circadian rhythm in PER2::LUC bioluminescence. The circadian rhythm in PER2::LUC in the retina is likely to be generated within the inner retina since the PER2::LUC bioluminescence signal is present only in the inner retina. Finally, a preliminary study by Besharse et al. [23], also using PER2::LUC knockin mice, reported that the cornea, the iris, and RPE-choroid may also express circadian rhythms in luciferase activity and in *Per2* mRNA. Our data are consistent with these results (Figure 1D and Figure 2C) and expand on the previous investigations by showing that *Per2* gene expression is under circadian control in the RPE in vivo, and that isolated RPE-choroid explants can express a long-lasting circadian rhythm in PER2::LUC bioluminescence. The circadian clock that controls this rhythm is likely to be located in the RPE cells since the choroid lacking the RPE does not emit measurable amounts of bioluminescence.

Previous studies have shown that PER2::LUC bioluminescence in vitro and *Per2* mRNA expression in vivo show a similar phase in many of the tissues/organs investigated [7]. Our data indicate that this is the case for the retina, but not for the RPE (Figure 1 and Figure 2). The reason for this discrepancy is not known, but we propose two plausible explanations. First, a retinal factor may be controlling the phase of the circadian oscillation in the RPE, and once this tissue and the retina are separated, the RPE may reset its circadian phase to a different phase. Alternatively, the culture procedure may be responsible for the discrepancy; in fact, a previous study has shown that in some tissue, the peak phases of the cultures can be particularly sensitive to some aspects of the culture procedure [24]. Further studies will be required to clarify this issue.

Earlier works have reported the presence of at least two photopigments in the RPE, including the newly discovered melanopsin [25,26], and it has been suggested that the RPE is directly photosensitive [26]. Our data indicate that the PER2::LUC bioluminescence circadian rhythm and amplitude are not affected by a light pulse, suggesting that the circadian clock in the RPE-choroid is not directly capable of light perception. Therefore, it can be speculated that the light entrainment of the circadian rhythm of the PER2::LUC bioluminescence rhythm in the RPE-choroid may depend on the retinal photoreceptor. The mechanism by which this information is transmitted from the photoreceptor to the RPE is unknown, but it may involve diffusible factors such as melatonin and dopamine, two well known modulators of retinal circadian rhythms [1].

Previous studies have also shown that the circadian rhythm of disc shedding in the rat retina persist after resectioning of the optic nerve or lesioning of the SCN [13,15], demonstrating that this circadian rhythm is controlled by a circadian clock within the eye. However, this rhythm could not be entrained to a new light cycle after the optic nerve was severed, suggesting that some component of the circadian regulation of disc shedding may be under the control of the SCN [13]. Our data indicate that the PER2::LUC bioluminescence rhythm in the RPE-choroid is not affected by SCN lesions, implying that the mechanisms controlling PER2::LUC bioluminescence in the mouse RPE-choroid reside within the eye. However, it is important to mention that these data do not define the contribution of the SCN to the regulation of the circadian rhythms in the RPE in vivo.

In conclusion, our data demonstrate that a circadian rhythm in PER2::LUC bioluminescence can be recorded from cultured RPE; light does not phase-shift the circadian rhythm in the RPE; and SCN lesions do not affect the phase of this rhythm. We believe that these results indicate the value of designing studies aimed at elucidating the molecular mechanisms responsible for the regulation of the rhythmic event in the RPE.
